# Predictors of blood glucose change and vascular complication of type 2 diabetes mellitus patients in Felege Hiwot Referral Hospital, North West Ethiopia

**DOI:** 10.1038/s41598-021-92367-w

**Published:** 2021-06-21

**Authors:** Nigusie Gashaye Shita, Essey Kebede Muluneh

**Affiliations:** 1 Department of Statistics, Debre Markos University, Debre Markos, Ethiopia; 2 School of Public Health, Bahir Dar University, Bahir Dar, Ethiopia

**Keywords:** Biomarkers, Endocrinology, Risk factors, Mathematics and computing

## Abstract

Vascular complication results in serious physical damages which may lead to the death of Type 2 diabetes mellitus patients. Studying the determinant factors of changes in blood glucose level and duration of time to the development of vascular complications helps to save the lives of citizens. A retrospective cohort study was conducted among type 2 diabetes mellitus (T2DM) patients enrolled between December 2011 and December 2012 at Felege Hiwot Referral Hospital. A total of 159 T2DM patients were included in the study. Joint modelling of longitudinal and survival analysis was employed to identify predictors of Blood Glucose Change and Vascular Complication of Type 2 Diabetes Mellitus Patients. The prevalence of vascular complication in Type 2 diabetes patients was 23.3%. Half of these patients developed an avascular complication after 24 months from the onset of the follow-up. The significant predictors of shorter time to development of vascular complication were positive proteinuria (adjusted hazard ratio (AHR) = 1.62, CI: 1.08–2.41), increase in the level of serum creatinine (AHR = 4.12, CI: 1.94–8.74), cholesterol ≥ 200 mg/dl (AHR = 1.54, CI: 1.01–2.35), and log (fasting blood glucose) (AHR = 1.453, CI: 1.004–2.104). The predictors of progression of fasting blood glucose were duration of treatment (CL: − 0.015, − 0.0001), hypertension (CL: 0.018, 0.098), baseline fasting blood glucose level 126–139 and 140-199 mg/dl (CI: − 0.40, − 0.31) and (CI: − 0.24, − 0.17**)**, respectively. Male T2DM patients, patients with more visits to the hospital and patients who required one oral agent had a relatively lower progression of blood sugar level. Type 2 diabetes mellitus patients having higher cholesterol level, positive proteinuria, higher fasting blood sugar and a lesser number of hospital visits had a higher risk of developing a complication.

## Introduction

Diabetes mellitus is a metabolic disorder of multiple etiologies characterized by chronic hyperglycemia with disturbance in carbohydrate, fat and protein metabolism resulting from defects in insulin secretion, insulin action or both^1^.

IDF reported that the worldwide prevalence of diabetes was estimated to be 8.8% (7.2–11.3%) in 2017 affecting 424.9 (346.4–545.4) million adults aged 20–79, including 212.4 million who are undiagnosed. There were approximately 4.0 (3.2–5.0) million people estimated to have died due to diabetes worldwide in the same year. It is projected that by 2045 there will be 628.6 (477–808.7) million people living with diabetes.

In the African region with 69.2%, undiagnosed diabetes has a prevalence of 3.3%^2^ whereas in Ethiopia the number is estimated at1 to 10 million in 2015^3^.

Diabetes mellitus has emerged as one of the rapidly increasing non-communicable diseases and a major public health challenge in developing countries like Ethiopia with a consequence of Chronicity and complications like disability and premature death^4^ due to long-term effects of untreated diabetes mellitus^5,6^. Hence, diabetes mellitus patients with hyperglycemia for a long period are highly prone to diabetic complications and mortality worldwide^2,7^.

Studies in Ethiopia showed that the incidence of vascular complications among Type 2 diabetes mellitus patients was found to be 40.6%^8^ whereas the prevalence of diabetic nephropathy and retinopathy were 6.1% and 41.4% respectively^9,10^.

Prior studies have not used the joint modelling approach of longitudinal and survival data analysis for blood glucose change and vascular complications among patients with Type 2 diabetes mellitus patients. Sometimes, the interest of researchers may lie in the association between the longitudinal process (longitudinal change of the blood glucose level) and survival process (time to develop complications of diabetes mellitus). A separate analysis, however, could not serve the purpose. Joint models of longitudinal and survival data have attracted increasing attention over the last two decades. Joint models of longitudinal and survival data incorporate all information simultaneously and provide valid and efficient inferences^11–13^. Hence, this study aimed to identify predictors of blood glucose change and time to vascular complications among patients with Type 2 diabetes using the joint modelling approach of longitudinal and survival data analysis.

## Results

### Characteristics of study participants

Out of a total of 159 newly diagnosed T2DM patients, 23.3% developed vascular complications. The incidences of retinopathy, nephropathy, neuropathy, stroke, CHD, and PAD were 18.9, 13.5, 40.5, 5.4, 8.1, and 13.5 cases per 100-person year of observation respectively. The overall mean and median estimated survival time of patients under the study was 24.77 and 20 months respectively (Table 1).

**Table 1:** Descriptive statistics for baseline continuous variables of T2DM patients under Anti diabetes drug in FHRH, December 2011–March 2016.

Status of patient	Continuous variables	Mean	Std. dev	Minimum	Maximum	Median	Q1	Q3
No events	Time in month	24.49	18.78	2	66	18.62	8	42
	Age in year	52.12	12.61	25	80	50.5	43	62
	Number of hospital visits	10.89	10.42	2	45	7	3	15
	Weight in kg	70.4	3.9	69.5	68	72	65	82
	Creatinine in mg/dl	1.01	0.22	0.34	1.82	1.04	0.9	1.1
Any one of vascular complication	Time in months	25.69	17.11	1.7	65	24	13	37.33
	Age in years	50.03	11.03	28	80	50	42	58
	Number of hospital visit	10.59	10.37	2	43	7	4	11
	Weight in kg	71.9	10.6	76	62	81	58	87
	Creatinine in mg/dl	1.12	0.24	0.6	1.59	1.04	1.04	1.3
Over all	Time in month	24.77	18.36	1.7	66	20	8	40.67

### Demographic variables

There were more females patients (59.75%) than males (40.25%). Generally, male T2DM patients had a 0.043 mg/dl lower FBS level compared to female T2DM patients (Table 4). The mean (SD) age for patients at the start of treatment was 51.6 (± 12.6) years (Table 1).

**Figure 1:**
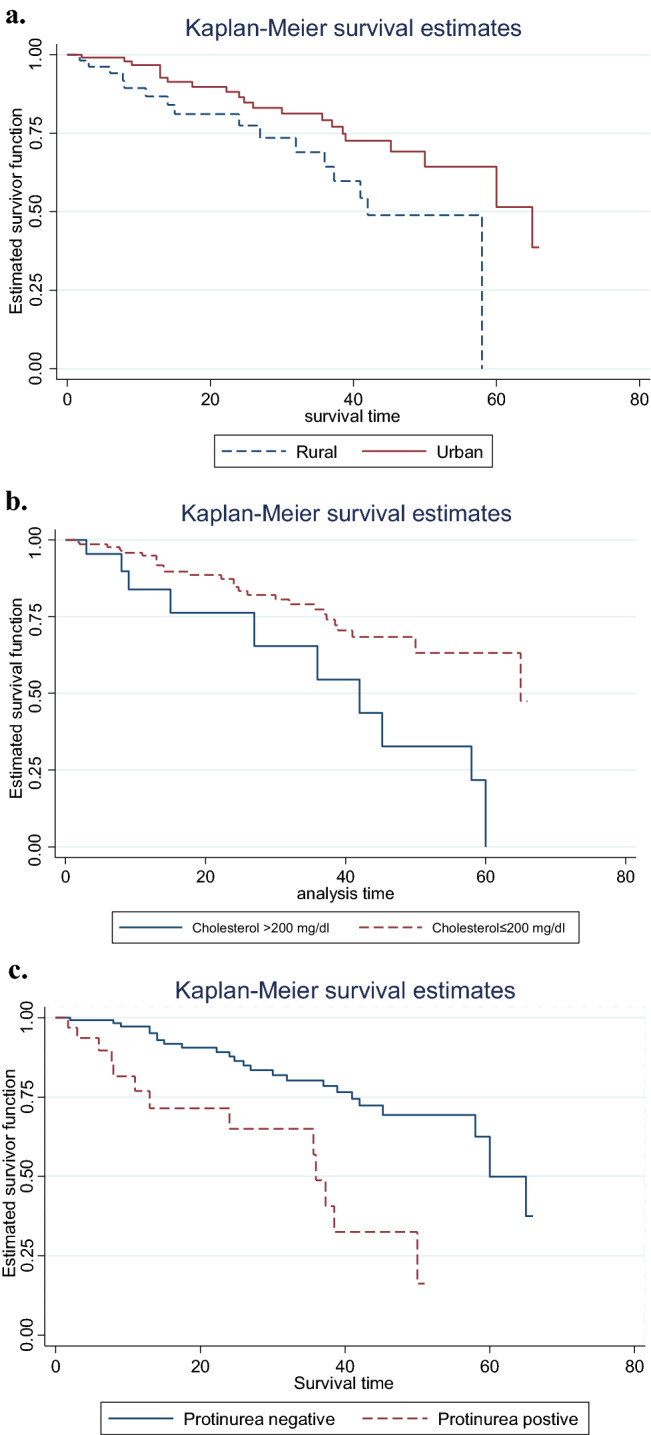
** (a–c)** Plots of Kaplan–Meier survival functions of categorical variables among T2DM Patients under Anti diabetes treatment at FHRH, December 2011–March 2016.

Patients who reside in rural represented 66.04% of the study sample. And, it had a significantly shorter time to develop vascular complications than that of urban dwellerT2DM patients (Table 2, Fig. 1a–c).

**Table 2:** Results of the Log-rank test for the categorical variables of T2DM patients under anti type 2 diabetes drugs in FHRH, December 2011–March 2016.

Factors	DF	Chi-square	P value
Gender	1	1.81	0.1789
Residence	1	4.97	0.0258
Proteinuria	1	16.58	0.0000
Treatment	4	0.78	0.6766
Hypertension co morbidity	1	0.67	0.4134
Cholesterol (mg/dl)	1	7.40	0.0065
Baseline FBS	2	0.57	0.7509

### Clinical variables

The mean (SD) weight for patients who didn’t develop vascular complication was 70.4 (± 3.9) kilograms whereas the average (SD) weight for patients who developed vascular complication was 71.9 (± 10.9) kg (Table 1).

Forty-two per cent of the patients had a developing hypertension history and had higher mean fasting blood glucose levels (185.82 mg/dl) which are higher than the mean fasting blood glucose levels of those with no hypertension history (185.7 mg/dl) (Table 3). In the main, T2DM Patients who developed hypertension had a 0.058 mg/dl higher FBS level compared to those who did not develop hypertension history (Table 4).

**Table 3:** Population characteristics and Univariate associations of factors with log of fasting blood glucose in FHRH, December 2011–March 2016.

Variables	Any one of Vascular complication	Mean	Std. dev	Minimum	Maximum	P value
**Gender**						
Female Male	64 (18.75%) 95 (26.30%)	191.84 181.25	64.54 66.07	88 80	377 381	0.1214
**Residence**						
Rural Urban	105 (20%) 54 (29.60%)	194.47 182.03	76.88 59.83	80 89	381 376	0.063
**Cholesterol (mg/dl)**						
> 200 ≤ 200 (ref)	22 (45.45%) 137 (19.70%)	210.28 182.21	72.61 63.78	88 80	377 381	0.0109
**Hypertension history**						
Yes No	69 (15.95%) 90 (28.90%)	185.82 185.70	67.40 64.46	89 80	377 381	0.0115
**Proteinuria**						
Positive Negative	127 (18.90%) 32 (40.60%)	186.28 181.28	67.93 64.95	94 80	376 381	0.733
**Baseline fasting blood glucose**						
126–139 mg/dl 140–199 mg/dl ≥ 200 mg/dl	26 (23.10%) 60 (25%) 73 (21.90%)	157.46 165.89 216.77	58.40 48.45 70.85	80 88 80	377 354 381	< 0.0001
**Treatment**						
Insulin alone or insulin plus oral agents More than one oral agent (no insulin) One oral agent	24 (16.67%) 24 (25%) 111 (24.32%	204.14 199.45 71.42	68.07 63.74 62.11	88 93 80	376 377 381	0.0002
Weight	–	70.71	1.25	58	87	0.138
Serum creatine	–	1.04	0.27	0.34	3	0.233

**Table 4:** Result of joint longitudinal and survival analysis on T2DM patients under anti type 2 diabetes drugs in FHRH, December 2011–March 2016.

	Covariates	Estimate	Standard error	95% confidence level		P value
				LCI	UCI	
Longitudinal process	Intercept	5.522	0.033	5.457	5.5859	< 0.0001
	Time	−0.0075	0.0038	−0.015	−0.0001	0.0482
	**Baseline FBS**					
	126–139 mg/dl 140–199 mg/dl ≥ 200 mg/dl (ref)	−0.4427 –0.2380	0.0359 0.0225	−0.5131 −0.2822	−0.3723 −0.1939	< 0.0001 < 0.0001
	Number of hospital visit	−0.0059	0.0010	−0.0079	–0.0039	< 0.0001
	**Hypertension history**					
	Yes No (ref)	0.0561	0.0195	0.0018	0.0942	0.0039
	**Gender**					
	Male Female (ref)	−0.0435	0.0207	−0.084	−0.003	0.0354
	**Residence**					
	Urban Rural (ref)	−0.0419	0.0224	−0.0857	0.0019	0.0610
	**Treatment**					
	More than one oral agent (no insulin) One oral agent Insulin alone or insulin plus oral agents (ref)	−0.0155 −0.0871	0.0286 0.0267	−0.0715 −0.1394	0.0405 −0.0347	0.5878 0.0011
Event process	Intercept	7.6006	1.3708	4.9139	10.2874	< 0.0001
	Number of hospital visit	0.0463	0.0109	0.0249	0.0678	< 0.0001
	Serum creatine	−1.4148	0.3844	−2.1682	−0.6614	0.0002
	**Proteinuria**					
	Positive Negative (ref)	−0.4793	0.2039	−0.8790	−0.0796	0.0188
	**Cholesterol (mg/dl)**					
	> 200 ≤ 200 (ref)	−0.4315	0.2154	−0.8536	−0.0095	0.0451
	**Treatment**					
	More than one oral agent (no insulin) One oral agent Insulin alone or insulin plus oral agents (ref)	−0.6080 −0.5272	0.3804 0.3200	−1.3535 −1.1544	0.1375 0.0999	0.1099 0.0994
	Association	−0.3737	0.1889	−0.7439	−0.0035	0.0478
	log (shape)	0.6400	0.1234			< 0.0001

Formula calculating change in FBS for longitudinal process =  (${e}^{estimate}$-1), formula calculating adjusted hazard ratio for event process $={e}^{-estimate}$.

*ref* = reference group.

Insulin alone or insulin plus oral agents, users comprised 15.1% of the study population and had higher mean fasting blood glucose values (204.14 mg/dl) than multiple oral medication users (199.45 mg/dl) and that one oral agent users (171.42 mg/dl) (Table 3). In general, patients who required one oral agent had a 0.0834 mg/dl lower FBS compared to those who used insulin alone or insulin plus oral agents (Table 4).

The Minimum, Maximum, and median value for the number of hospital visit was found to be 2, 45, and 7 (IQR = 3–15) respectively whereas, for every one-day increase in the number of hospital visits per follow up period, the hazard of developing vascular complication value was 0.95 months lower.

On average, for every one-day increase in the number of hospital visits per follow up period, the FBS value gets lower by 0.006 mg/dl. On average, for every six-month increase in the duration of the Anti T2DM treatment, the FBS value decreases by 0.007 mg/dl (Table 4).

### Physiological characteristics

Patients with baseline fasting blood glucose of 200 mg/dl or more comprised 45.9% of the study population. And, it had higher mean fasting blood glucose values (216.77 mg/dl) compared to those with baseline fasting blood glucose 140–199 mg/dl (165.89 mg/dl) and 126–139 mg/dl (157.46 mg/dl) (Table 3). Generally speaking, Patients with baseline fasting blood glucose of 126–139 mg/dl and 140–199 mg/dl had a 0.358 mg/dl and 0.212 mg/dl lower FBS level respectively compared to those whose baseline fasting blood glucose of 200 mg/dl or more (Table 4).

The minimum and maximum fasting blood glucose levels of the patients were 80 and 381 mg/dl respectively under the study period. On average, for every one mg/dl increase *true unobserved* log FBS, the hazard of developing vascular complication value was 1.45 months higher (Table 4).

Patients with cholesterol levels more than 200 mg/dl represented 13.8% of the study sample and had higher mean fasting blood glucose values (210.28 mg/dl) than those with cholesterol levels 200 mg/dl or lower (182.03 mg/dl) (Table 3). Whereas, patients with a cholesterol level of 200 mg/dl or lower had a significantly longer time to develop a complication than that of T2DM patients with a cholesterol level of more than 200 mg/dl (Table 2, Fig. 1a–c). That is Patients with a cholesterol level greater than 200 mg/dl had a 1.54 times higher hazard of developing vascular complications compared to those patients with a cholesterol level of 200 mg/dl or lower (Table 4).

Patients with positive proteinuria represent 79.9% of the study sample. And, it had a significantly shorter time to develop vascular complications than those who had negative proteinuria patients (Table 2, Fig. 1a–c). To be precise, the risk of developing vascular complications for T2DM patients with positive proteinuria was 1.61 times more compared to those with negative proteinuria T2DM patients (Table 4).

The Minimum, Maximum, and median value for creatinine was found to be 0.34, 1.82, and 1.04 mg/dl (IQR = 0.9–1.1) respectively. On average, for every one mg/dl in increase serum creatinine, the hazard of developing vascular complication value was 4.12 times higher (Table 4).

## Discussion

In this study, survival-longitudinal sub-model analysis was used to identify the determinant factors that affect the time to develop vascular complication and changes in the blood glucose level. The variables gender, hypertension history, number of hospital visits, baseline FBS and treatment were found to have a significant association with the progression of FBS level. On the other hand, serum creatinine, proteinuria, cholesterol level, number of hospital visits and FBS were found to have a significant association with the hazard (risk) of developing vascular complications.

The progression (increase) of fasting blood glucose level of female T2DM patients was faster than male T2DM patients. This result contradicted the study conducted in Ghana^14^ which showed that the rate of change in blood glucose level for males was faster than the change in women. The possible reason for this finding might be that males were more drinker than the female which may accelerate the progression of blood glucose level. In the Ethiopian context males are more exposed to higher physical activities as compared to female subjects which result in improved insulin sensitivity, decrease blood glucose and blood pressure level, weight loss, reduce triglycerides and cholesterol, increase muscle tone, improve circulation, stress relief and well-being feelings^15^.

Patients who required one oral agent had a lower progression change of FBS compared to those who used insulin alone or insulin plus oral agents. Insulin use is also a factor of disease in severity and was a predictor of poorer glycemic control in this study. They also found insulin users to have poorer glucose control. This study is consistent with a study conducted by Benoit and his colleagues^16^.

The hazard of vascular complication for T2DM patients with a cholesterol level greater than 200 mg/dl was higher as compared to T2DM patients with a cholesterol level of 200 mg/dl or lower. This study is in line with the study done in Iran, which showed that higher levels of cholesterol were positively associated with the risk of vascular complications^17^.

The hazard of vascular complications increases with increasing fasting blood glucose level. This is consistent with a study done on the association of the development of vascular complication on fasting blood glucose in T2DM conducted^18^ and another study on the association of complication and glycemic conducted in the United Kingdom Prospective Diabetes Study 33 and 34^19,20^. Both studies reported that the hazard of vascular complication has a positive association with fasting blood glucose.

This study assumed that all the vascular complications are caused by diabetes mellitus and considered vascular complication as a composite outcome for stroke, coronary heart disease, peripheral arterial disease, retinopathy, nephropathy, and neuropathy. This may overestimate the rate of vascular complication. Besides, the limitation of the study is the limited information on important predictors such as family history, BMI, and the type of interventions, including the type of exercises and nutritional status of a patient that may have influenced the outcome variables. Due to *a lack* of data on these potential predictors for most of the patients involved in the study, we were unable to include them in the analyses. Therefore, more public health and epidemiology researches are needed to examine the impact of these variables on population health in general and in particular, people living with T2DM to avoid its complications over time and to identify new risk factors for T2DM.

## Conclusions and recommendations

The prevalence of vascular complication of Type 2 Diabetes patients in this study was 23.3%. Half of the patients in the study developed any form of vascular complication after 24 months from the onset of the follow-up time**.**

The progression of the fasting blood glucose level of female T2DM patients was faster than male T2DM patients. Patients who required one oral agent had a lower progression change of FBS compared to those who used insulin alone or insulin plus oral agents. Patients with more visits to the hospital have a relatively lesser progression rate of blood sugar level.

Type 2 diabetes mellitus patients having higher cholesterol level, positive proteinuria, higher fasting blood sugar and a lesser number of hospital visits have a higher risk of developing a complication.

In light of these findings, health professionals in the DM follow up clinics should give targeted intervention for type 2 DM patients with positive proteinuria, cholesterol level greater than 200 mg/dl, with higher serum creatinine levels and fasting blood glucose levels to maximize efforts on the prevention of T2DM complication and risk minimization of vascular complication.

## Methods

### Study design and period

An Institution-based retrospective follows up study design was used. Records of newly diagnosed type 2 diabetes mellitus (DM) patients who were enrolled between December 2011 and December 2012 were selected and followed continuously until January 2016.

### Study area and study population

This study was conducted among type 2 DM patients at Felege Hiwot Referral Hospital (FHRH). FHRH is found in Bahir Dar, the capital city of the Amhara Regional State, a region in the Northwest of Ethiopia. The study population was all type 2 diabetic patients aged 18 years or older who came to the hospital for diagnosis and follow up from December 2011 to December 2012. These patients were followed until January 2016. Patients who were free from any of the vascular complications at the start of treatment and patients with at least two observations (follow-ups) within the study period were included in the analyses leading to a total of 159 patients and 888 observations.

### Data collection procedures and data quality control

The study used secondary data obtained from the patients’ files. A data extraction checklist was prepared to collect the data and the reviewed records were identified by their medical registration card number. Both the longitudinal and survival data were extracted from the patient's chart. The primary outcome was having any of the vascular complications such as retinopathy, nephropathy, neuropathy, stroke, peripheral arterial disease and coronary heart disease. These complications were determined based on the clinical decision of the physician. Diabetic retinopathy was defined by both direct and indirect ophthalmoscope assessments done by retinal specialists confirmed by fundus photography. Neuropathy was defined by a history of numbness, paraesthesia, tingling sensation confirmed by touch sensation by 10 g monofilament, vibration sense by biothesiometer and ankle reflex. Nephropathy was defined as worsening of blood pressure control, swelling of the foot ankle, hands or eyes, increased need to urinate, protein in the urine with a confirmation by tests like blood test, urine test, renal function test and imaging test. Stroke is defined as patients with sudden difficulty in speech and comprehension, sudden paralysis or numbness of the face, arm or leg, sudden trouble with walking and confirmation imaging with computerized tomography scan or magnetic resonance imaging. The Peripheral arterial disease was defined by a history of intermittent claudication, coldness in the lower extremities (especially when compared with the other side), and weak or absent peripheral pulses in the lower extremities and confirmation via Doppler ultrasound. Coronary heart disease was diagnosed by symptoms of angina, shortness of breath, a crushing sensation in the chest, and pain in the shoulder or arm and sweating. Additionally, CHD was confirmed by electrocardiogram or echocardiogram^8,21,22^.

Both baseline and time-dependant characteristics were assessed from the patient’s registration document. The first characteristic assessed was the demographic variables such as age, gender and residence. The second characteristic assessed was the clinical variables such as hypertension comorbidity, which was defined as a history of antihypertensive drug use or SBP ≥ 140 mmHg or DBP ≥ 90 mmHg^8,23^, weight and type of treatment of DM. The third characteristic assessed was the physiological component such as creatinine, fasting blood sugar, systolic blood pressure, diastolic blood pressure, total cholesterol, which, were categorized as high and low^8,24,25^ and protein urea which was defined as positive if the urine albumin concentration is between 30 mg (mg)/24 h and 300 mg/ 24 h and negative if it is < 30 mg/24 h.

These data were collected by two nurses who had experience working with diabetic patients on follow-up. To control the data quality, training was given to the data collectors and their supervisor. The data extraction checklist was pre-tested for consistency of understanding the review tools and completeness of data items. The necessary adjustments were made on the final data extraction format and the filled formats were checked daily by the supervisor.

### Ethics approval and consent to participate

Ethical approval to conduct the study and human subject’s research approval for this study was received from Bahir Dar University, College of Sciences, Research Ethics Committee and the medical director of the Hospital. We confirm that all methods were performed by the relevant guidelines and regulations. As the study was retrospective, informed consent was not obtained from the study participants, but data were anonymous and kept confidential.

### Data analysis

Descriptive statistics were used to describe the percentage and frequency of the patients in the different categories of factors. Kaplan**–**Meier survival function and log-rank test were used to estimate and compare the survival experiences among the different groups of subjects respectively. The independent t-test or One-way ANOVA was used to assess significant differences in mean fasting blood glucose. Besides, the least significant differences method was used to assess individual differences.

### Joint models

Joint models were used to identify factors that determine the change of blood glucose level over time and duration of time until the occurrence of complications by analysing the repeated measure of fasting blood sugar (FBS) values and time to vascular complication simultaneously. The joint models consist of two linked sub-models, known as the longitudinal sub-model, and the survival sub-model.

### Longitudinal sub model

**Figure 2:**
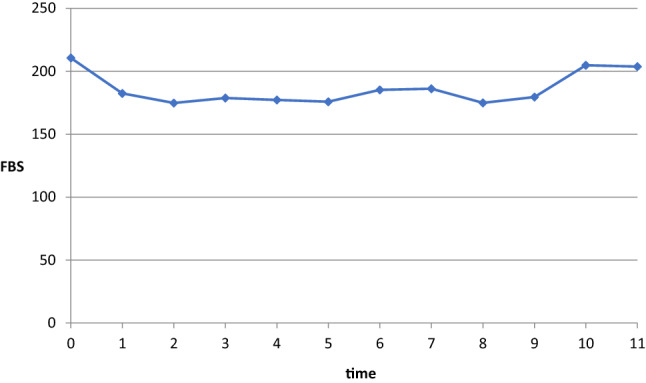
Fluctuation in mean FBS values over 6 months in FHRH, BD, 2016.

A linear mixed sub-model was used to assess the determinant factors that affect the progression of blood glucose level by analyzing the repeated measures data, FBS values. The observed mean FBS level profile of patients in Fig. 2 shows that the linearity assumption is not reasonable. Therefore, the analysis has to account for the longitudinal data structure and the observed nonlinearity of the FBS level estimated by log-transformed in the mixed model framework. The linear mixed sub-model can be rewritten as,

1 $$\begin{array}{c}{{y}_{i}\left (t\right)=m}_{i}\left (t\right)+{u}_{i}\left (t\right)+{\epsilon }_{i}\left (t\right)\\ {m}_{i}\left (t\right)={x}_{i}\left (t\right)\beta +{z}_{i}\left (t\right){b}_{i}\\ {b}_{i}\sim N\left (0,{\mathrm{\Sigma }}_{b}\right)\\ {\epsilon }_{i}\sim N (0,{\sigma }^{2}{I}_{ni})\end{array}$$

where y is an $n\times 1$ observational of FBS values, $\beta$ is a $p\times 1$ vector of unknown constants of fixed effects of the model, $X$ is an $n\times p$ known matrix of fixed effects associated with $\beta$, Z is an $n\times q$ known design matrix of random effects,${b}_{i}$ is a $q\times 1$ vector of unknown random effects, and ${\epsilon }_{i}\left (t\right)$ is an n $\times 1$ vector of error terms. Since the FBS values taken from a patient at different follow up times are assumed serially correlated, the stochastic term ${u}_{i}\left (t\right)$ is used to capture the remaining serial correlation in these observed measurements, not captured by the random effects^13^. The stochastic term is considered as a zero-mean stochastic process, independent of ${b}_{i}$ and ${\epsilon }_{i}\left (t\right)$

### Survival sub model

The survival sub-model was used to identify factors that affect the time taken until a T2DM patient develops some form of vascular complication. The survival sub-model has the form:

2 $$\log T_{i} = \mu + \alpha _{1} w_{{1i}} + \alpha _{2} w_{{2i}} + \cdots + \alpha _{p} w_{{pi}} + \theta m_{i} \left ( t \right) + \sigma \varepsilon _{i}$$

where $\mu$ is the intercept, ${w}_{ji}$ denote the jth baseline covariates of the ith observation with a corresponding vector of regression coefficients ${\alpha }_{j} (j=\mathrm{1,2},\cdots \cdots \cdots ,p)$, ${T}_{i}$ denotes the observed failure time for the ith subject $(i=\mathrm{1,2},\cdots \cdots \cdots ,n)$, ${m}_{i}\left (t\right)$ is the unobserved value of the longitudinal outcome at the time $t$, $\sigma$ is the scale parameter, and ${\epsilon }_{i}$ denote the ith observation error terms having a standard probability distribution. Specifically, for this study, log-logistic distribution is an appropriate probability distribution than others.

### Parameter estimation for joint modelling

Restricted maximum likelihood estimation was used to estimate the model parameters. Maximum likelihood estimation for joint models is based on the maximization of the log-likelihood corresponding to the joint distribution of the time-to-vascular complication due to T2DM and longitudinal outcomes, (${T}_{i},{\delta }_{i},{y}_{i}$). Then, the overall log-likelihood for all the observed data is formulated as,

3 $${\mathrm{L}}\left (\theta \right)=\sum _{j}P\left ({T}_{i},{\delta }_{i},{y}_{i};\theta \right)$$

The maximization of a function (Eq. 3) with respect to $\theta$ requires a combination of numerical integration and optimization algorithms because both the integral concerning the random effects in the probability distribution of longitudinal outcomes and survival function in the probability distribution of time to vascular complication due to T2DM do not have an analytical solution. We used pseudo adaptive Gauss Hermite numerical integration techniques to obtain the approximate solution, and the baseline hazard function follows a Weibull distribution.

To build both separate longitudinal and survival analysis the procedure we followed is first we fit a univariable model for each of the explanatory variables and based on statistical significance identifies variables to be candidates for the multivariable analysis. As naturally different factors/variables do not operate separately, multivariable analysis helps to control for confounders and analyze the effects of a factor in the presence of other factors in the model.

After we have applied the above model-building strategies, Longitudinal and survival sub-models were fitted by joining separated longitudinal and survival analysis using a JM package of R 3.4. We used Akaike and Bayesian information criteria to select the appropriate joint models, and the model with the smallest AIC or BIC was considered the best fit^26,27^.

### Ethics approval and consent to participate

Ethical approval to conduct the study and human subject research approval for this study was received from Bahir Dar University, College of Sciences, Research Ethics Committee and the medical director of the Hospital. As the study was retrospective, informed consent was not obtained from the study participants, but data were anonymous and kept confidential.

## Data Availability

The data sets analysed in this study available from the corresponding author on reasonable request.

## Abbreviations

ADA: America Diabetes Association
AHR: Adjusted hazard ratio
AIC: Akaike information criteria
ANOVA: Analysis of variance
BD: Bahir Dar
BIC: Bayesian information criteria
BP: Blood pressure
CL: Confidence interval
CHD: Coronary heart disease
df: Degree of freedom
FBS: Fasting blood glucose
FHRH: Felege Hiwot Referral Hospital
LUC: Lower confidence limit
OPD: Outpatient department
PAD: Peripheral arterial disease
P-value: Probability value
Q1: Quartile 1
Q3: Quartile 3
Std. dev: Standard deviation
T2DM: Type 2 diabetes mellitus
UCL: Upper confidence limit
UKPDS: United Kingdom Prospective Diabetes Study
